# Drug Metabolism and Pharmacokinetics of Antisense Oligonucleotide Therapeutics: Typical Profiles, Evaluation Approaches, and Points to Consider Compared with Small Molecule Drugs

**DOI:** 10.1089/nat.2022.0054

**Published:** 2023-03-30

**Authors:** Hideo Takakusa, Norihiko Iwazaki, Makiya Nishikawa, Tokuyuki Yoshida, Satoshi Obika, Takao Inoue

**Affiliations:** ^1^ Drug Metabolism & Pharmacokinetics Research Laboratories, Daiichi Sankyo Co., Ltd., Tokyo, Japan.; ^2^ Sohyaku. Innovative Research Division, Mitsubishi Tanabe Pharma Corp., Yokohama, Japan.; ^3^ Faculty of Pharmaceutical Sciences, Tokyo University of Science, Noda, Japan.; ^4^ Division of Molecular Target and Gene Therapy Products, National Institute of Health Sciences, Kawasaki, Japan.; ^5^ Graduate School of Pharmaceutical Sciences, Osaka University, Suita, Japan.

**Keywords:** antisense oligonucleotide, drug metabolism, pharmacokinetics, biodistribution, bioanalysis

## Abstract

Oligonucleotide therapeutics are attracting attention as a new treatment modality for a range of diseases that have been difficult to target using conventional approaches. Technical advances in chemical modification and drug delivery systems have led to the generation of compounds with excellent profiles as pharmaceuticals, and 16 oligonucleotide therapeutics have been marketed to date. There is a growing need to develop optimal and efficient approaches to evaluate drug metabolism and pharmacokinetics (DMPK) and drug–drug interactions (DDIs) of oligonucleotide therapeutics. The DMPK/DDI profiles of small molecule drugs are highly diverse depending on their structural and physicochemical characteristics, whereas oligonucleotide therapeutics share similar DMPK profiles within each chemistry type. Most importantly, the mechanisms and molecules involved in the distribution and metabolism of oligonucleotides differ from those of small molecules. In addition, there are considerations regarding experimental approaches in the evaluation of oligonucleotides, such as bioanalytical challenges, the use of radiolabeled tracers, materials for *in vitro* metabolism/DDI studies, and methods to study biodistribution. In this review, we attempt to summarize the DMPK characteristics of antisense oligonucleotide (ASO) therapeutics and discuss some of the issues regarding how to optimize the evaluation and prediction of the DMPK and DDI of ASOs.

## Introduction

Oligonucleotide therapeutics are gaining attention as a new drug modality to reach therapeutic targets that cannot be treated using existing drug modalities, such as genetic diseases [1,2]. Early developments of oligonucleotide therapeutics were plagued by poor stability in the body and inadequate delivery to target tissues, but advances in nucleotide chemical modifications and targeted delivery system have generated drug-like compounds with better efficacy, safety, and pharmacokinetic profiles [3–5]. The research and development of oligonucleotide therapeutics accelerated after the successful development of mipomersen as the first systemically administered drug in this class in 2013. As of August 2022, 16 oligonucleotide therapeutics had been approved, as summarized in Table 1.

**Table 1.** List of Approved Oligonucleotide therapeutics

Generic name	Type	Modification	Approval	Target	Indication	RoA
Fomivirsen	Antisense	PS	US 1998 EU 1999	CMV IE2 mRNA	CMV retinitis	IVT
Pegaptanib	Aptamer (PEG)	2′-F 2′-OMe	US 2004 EU 2006 JP 2008	VEGF165 (protein)	Neovascular ARMD	IVT
Mipomersen	Antisense (gapmer)	PS 2′-MOE	US 2013	ApoB-100 mRNA	HoFH	SC
Eteplirsen	Antisense (SSO)	PMO	US 2016	Dystrophin pre-mRNA	DMD	IV
Nusinersen	Antisense (SSO)	PS 2′-MOE	US 2016 EU 2017 JP 2017	SMN2 pre-mRNA	Spinal muscular atrophy	IT
(CpG1018)^1)^	CpG oligomer	PS	US 2017 EU 2021	TLR9 (protein)	HBV infection	IM
Inotersen	Antisense (gapmer)	PS 2′-MOE	US 2018 EU 2018	TTR mRNA	hATTR	SC
Patisiran	siRNA (LNP)	2′-OMe	US 2018 EU 2018 JP 2019	TTR mRNA	hATTR	IV
Volanesorsen	Antisense (gapmer)	PS 2′-MOE	EU 2019	ApoCIII mRNA	FCS	SC
Givosiran	siRNA (GalNAc)	PS (partial) 2′-OMe; 2′-F	US 2019 EU 2020 JP 2021	ALAS1 mRNA	Acute hepatic porphyria	SC
Golodirsen	Antisense (SSO)	PMO	US 2019	Dystrophin pre-mRNA	DMD	IV
Viltolarsen	Antisense (SSO)	PMO	US 2020 JP 2020	Dystrophin pre-mRNA	DMD	IV
Lumasiran	siRNA (GalNAc)	PS (partial) 2′-OMe; 2′-F	US 2020 EU 2020	HAO1 mRNA	PH1	SC
Inclisiran	siRNA (GalNAc)	PS (partial) 2′-OMe; 2′-F	EU 2020 US 2021	PCSK9 mRNA	HeFH	SC
Casimersen	Antisense (SSO)	PMO	US 2021	Dystrophin pre-mRNA	DMD	IV
Vutrisiran	siRNA (GalNAc)	PS (partial) 2′-OMe; 2′-F	US 2022	TTR mRNA	hATTR	SC

1) CpG1018 is an oligonucleotide added as an adjuvant to the hepatitis B virus vaccine and is listed here as one type of oligonucleotide therapeutics.

ALAS1, delta-aminolevulinate synthase 1; ApoB, apolipoprotein B; ApoCIII, apolipoprotein CIII; ARMD, age-related macular degeneration; CMV, cytomegalovirus; DMD, Duchenne muscular dystrophy; EU, European Union; FCS, familial chylomicronemia syndrome; GalNAc, N-Acetylgalactosamine; hATTR, hereditary transthyretin amyloidosis; HAO1, hydroxyacid oxidase 1; HBV, hepatitis B virus; HeFH, heterozygous familial hypercholesterolemia; HoFH, homozygous familial hypercholesterolemia; IE2, immediate-early 2; IM, intramuscular; IT, intrathecal; IV, intravenous; IVT, intravitreal; JP, Japan; LNP, lipid nanoparticle; PCSK9, proprotein convertase subtilisin kexin 9; PEG, polyethylene glycol; PH1, primary hyperoxaluria type 1; RoA, route of administration; SC, subcutaneous; SMN2, survival motor neuron 2; TLR9, Toll-like receptor 9; TTR, transthyretin; US, United States; VEGF, vascular endothelial growth factor.

There is a growing need to develop optimal and efficient approaches to evaluate the drug metabolism and pharmacokinetics (DMPK) and drug–drug interactions (DDIs) of oligonucleotide therapeutics. Currently the DMPK/DDI profiles of oligonucleotide therapeutics are generally evaluated according to the concepts and methodologies applied to small molecule drugs, but it has become clear that the factors that characterize the DMPK profile of oligonucleotides, especially the mechanisms and molecules involved in their distribution and metabolism, differ from those for small molecules. In addition, there are considerations regarding the experimental approaches used in the evaluation of oligonucleotides, such as bioanalytical challenges, the use of radiolabeled tracers, materials for *in vitro* metabolism/DDI studies, and methods to study biodistribution. This review outlines the DMPK characteristics of antisense oligonucleotide (ASO) therapeutics and discusses the issues and perspectives regarding how to optimize the evaluation and prediction of the DMPK/DDI of oligonucleotides.

## Classification and Characteristics of Oligonucleotide Therapeutics

**FIG. 1.**
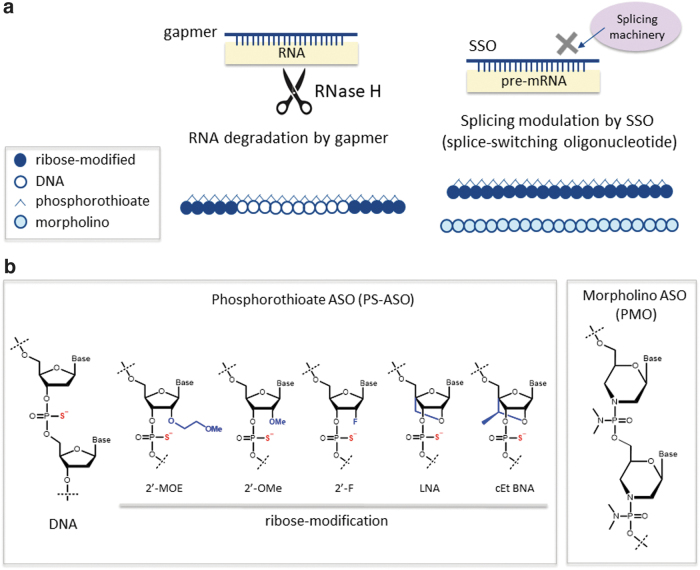
Classification and structures of ASO therapeutics: **(a)** Two major types of ASOs: gapmers and SSOs. **(b)** Chemical modifications used in ASOs to improve stability against nucleases and affinity to the target RNA. ASO, antisense oligonucleotide; SSO, splice-switching oligonucleotide.

Oligonucleotide therapeutics are generally considered to be “chemically synthesized drugs that consist of an oligonucleotide containing about one dozen to several dozen unmodified or modified nucleic acids and directly exert pharmacological actions without being translated into proteins.” DNA- and messenger RNA-based gene therapy products are also modalities composed of nucleic acids but differ from oligonucleotide therapeutics in that they are translated into proteins and are manufactured biologically or enzymatically. The main categories of oligonucleotide therapeutics are presented in Table 2 and shown in Fig. 1. Oligonucleotide therapeutics can be primarily classified into “RNA-targeting drugs” and “protein-targeting drugs.” Most of the approved oligonucleotide therapeutics (14 of 16 drugs) are RNA targeting, consisting of ASOs and small interfering (si) RNAs (Table 1).

**Table 2.** Classification and Characteristics of Major Oligonucleotide Therapeutics

	Antisense	siRNA	Aptamer	CpG oligo
Structure	ssDNA/RNA	dsRNA	ssDNA/RNA	ssDNA
Target	mRNA, pre-mRNA, miRNA	mRNA	Extracellular protein	Protein (TLR9)
Site of action	Nucleus, cytoplasm	Cytoplasm	Cell surface Extracellular site	Cell surface (Endosomal lumen)
Mechanism of action	RNA degradation, splicing switch, miRNA inhibition	mRNA degradation	Inhibition of protein function	Activation of innate immunity

ds, double-stranded; ss, single-stranded; TLR9, Toll-like receptor 9.

The ASOs currently used clinically exhibit one of two types of mechanisms of action [6–8] (Fig. 1a). One of these mechanisms involves the downregulation of target gene expression through the induction of RNaseH-dependent RNA degradation. ASOs with this mechanism of action are called gapmers and contain a central sequence of phosphorothioate (PS) DNA nucleotides flanked by sequences of sugar-modified residues at each end. The other mechanism of action involves modulation of splicing processes, caused by binding to a splicing regulatory region. ASOs with this mechanism of action are called splice-switching oligonucleotides (SSOs), and they upregulate functional target gene expression through exon skipping or inclusion. Oligonucleotide therapeutics that target proteins can be designed as aptamers and CpG oligonucleotides (CpG oligos), and one of each type has received regulatory approval [9,10].

The successful development of oligonucleotide therapeutics requires the oligonucleotide to be stable in the body and to bind potently to the target RNA; a wide variety of nucleotide modification techniques has been developed and used to meet these ends (Fig. 1b) [11–13]. Because oligonucleotides are primarily degraded through hydrolysis by nucleases in the body, stabilization of the phosphodiester backbone is required. One of the backbone modifications in the ASOs used clinically is a PS modification, in which an oxygen atom of the phosphodiester linkage is replaced with a sulfur atom. PS-modified oligonucleotides often also have a chemically modified sugar portion with 2′ substitutions such as 2′-O-methyl RNA (2′-OMe), 2′-O-methoxyethyl RNA (2′-MOE), 2′-fluoro RNA (2′-F), 2′-O, 4′-C-methylene bridged/locked nucleic acid (2′, 4′-BNA/LNA), or other cross-linking modification. Phosphorodiamidate morpholino oligomers (PMOs), made by substituting the phosphodiester bond of oligonucleotides with uncharged phosphorodiamidate bonds and introducing a morpholino ring instead of ribose, represent another commonly used chemical modification.

The ASOs currently under clinical development can be broadly classified as either PS-oligonucleotides, whose nucleic acids are all PS modified, or PMOs, whose nucleic acids are all morpholino nucleic acids. As discussed later, the DMPK profiles of oligonucleotide therapeutics are highly dependent on their chemical modifications.

## Absorption, Distribution, Metabolism, and Excretion-Related Profiles of ASO Therapeutics

In general, the behavior of a drug in the body is classified into the processes of its absorption from the site of administration to the systemic circulation; distribution from the circulating blood to various tissues; metabolism mediated by enzymes in the liver and other tissues to change its chemical structure; and excretion through the urine and feces in the terminal elimination phase. The absorption, distribution, metabolism, and excretion (ADME) profiles of siRNAs developed in combination with drug delivery systems (DDS) such as *N*-acetylgalactosamine (GalNAc) conjugation and lipid nanoparticles are highly affected by the nature of the drug delivery systems; however, the ADME profiles of ASOs have been well characterized and can be summarized by chemistry type [14–19]. In this section we describe the ADME-related profiles of ASO therapeutics and approaches for their evaluation compared with small molecule drugs.

### Absorption and plasma pharmacokinetics

“Drug absorption” generally refers to the intake of a drug from the site of administration to the bloodstream and is investigated when the clinical route of administration is not intravenous (e.g., oral or subcutaneous). The absorption profile of a drug depends on its solubility, membrane permeability, and other properties and is generally evaluated by measuring plasma concentrations of the drug. Plasma concentrations are determined using bioanalytical techniques such as liquid chromatography–mass spectrometry (LC/MS/MS) and are then used to calculate pharmacokinetic parameters such as area under the plasma concentration–time curve (*AUC*) and maximum plasma concentration. Bioavailability, which is calculated by comparing the *AUC* of a compound given through its clinical route of administration to that following intravenous administration, is an important indicator of how efficiently the compound is absorbed.

Orally administered PS-ASOs and PMOs are poorly absorbed because of their low membrane permeability resulting from their molecular weight and hydrophilicity [20]. Although oral delivery is not an option, unlike with small molecule drugs, the application of chemical modifications such as those described above allows systemic exposure to be achieved following intravenous or subcutaneous administration. Subcutaneously administered oligonucleotide therapeutics are rapidly absorbed with high bioavailability [21]. Local administration can be used for delivery to the eyes, central nervous system, and other targets not readily reached with systemic administration. Thus, the route of administration, chemical modifications, and drug delivery systems best suited to reaching the target tissue are important considerations for oligonucleotide development [3–5,22,23].

Plasma concentrations of absorbed oligonucleotide therapeutics decline rapidly, primarily because of tissue distribution, exhibiting a biphasic plasma pharmacokinetic profile. Once oligonucleotide therapeutics are distributed to the tissues, they tend to remain there for a long period, showing a slow decrease in tissue concentrations over time. Plasma concentrations in the elimination phase decrease in parallel with tissue concentrations, which can be explained by an equilibrium that is established between the tissue and plasma following distribution [24].

This phenomenon has been extensively studied and is demonstrated by the relationship between plasma and liver concentrations of 2′-MOE-modified PS-ASOs, including mipomersen [25]. In the case of mipomersen, its elimination half-life in monkey liver (34 days) is similar to that in monkey plasma (31.3 days), as well as in human plasma (31 days). The liver-to-plasma partition ratio of mipomersen at equilibrium in monkeys was calculated to be 5825, which is comparable to the value of 5861 observed in mice. In terms of the pharmacokinetics/pharmacodynamics (PK/PD) relationship of mipomersen, the plasma trough EC_50_ was determined to be 18 ng/mL in human ApoB transgenic mice, which is consistent with that reported in humans (10–17 ng/mL) [25,26]. These findings suggest that the liver-to-plasma partition ratio of mipomersen at equilibrium is similar across species, which is important for PK/PD translation from animals to humans and enables animals to be used as surrogates for liver exposure in humans.

A long terminal plasma half-life in equilibrium with tissue concentrations has also been reported for ASOs targeting the central nervous system (CNS). After intrathecal administration to monkeys, 2′-MOE PS-ASOs were rapidly distributed to CNS spinal cord tissues, with subsequent transfer to the systemic circulation, and exhibited long and comparable half-lives in the cerebrospinal fluid (t½: 107 days), plasma (t½: 110 days), and spinal cord tissues (t½: 97 days) [27].

Because plasma concentrations can be an important surrogate for tissue concentrations, highly sensitive bioanalytical methods need to be developed to determine low plasma concentrations during the elimination phase.

### Distribution

Distribution studies determine how a drug moves to and persists in tissues where it exerts its effect or causes toxicity. Drug distribution to the tissues throughout the body is generally evaluated by administration of radiolabeled drugs to animals. The amount of drug distributed to and persisting in various tissues is evaluated by quantitative whole-body autoradiography or the measurement of radioactivity in excised tissues over time after the administration of a radiolabeled drug. Pregnant animals are used to evaluate distribution to the placenta and fetus. Drugs in the blood can be bound to albumin and/or other plasma proteins, be present in red blood cells and/or other blood cells, or exist as a free form in the plasma. Because it is usually the free (unbound) form of a drug that can distribute to tissues and exert pharmacological activity, it is important to examine the protein-bound and unbound fractions of a drug in the plasma.

**FIG. 2.**
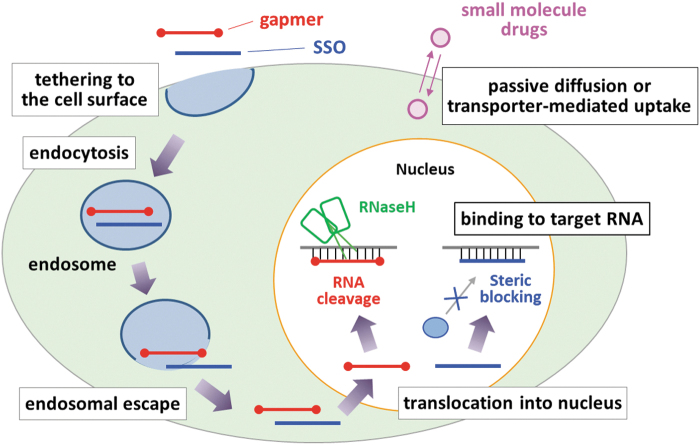
Schematic illustration of the cellular uptake and intracellular trafficking of ASOs.

Systemically administered oligonucleotide therapeutics are generally readily distributed to tissues with discontinuous or fenestrated capillaries, such as the liver and kidneys, whereas they are poorly distributed to the central nervous system, eyes, placenta, and other tissues with tight continuous capillaries. Because oligonucleotide therapeutics are not membrane permeable, they are taken up into cells by phagocytosis or receptor-mediated endocytosis rather than passive diffusion (Fig. 2) [24,28].

PS-ASOs are distinguished from PMOs by their protein-binding characteristics. The negatively charged backbone of PS-ASOs interacts with the hydrophilic regions of plasma proteins (primarily albumin), conferring overall high plasma protein binding [24,29,30]. Structural modifications of the ribose 2′ position change protein-binding affinity, and modifications with OMe, F, or LNA result in particularly high binding [30]. In contrast, electrically neutral PMOs tend to bind poorly to plasma proteins [12,17]. Because plasma protein binding is relevant to cellular uptake and glomerular filtration, the more persistent tissue distribution and slower urinary excretion profiles of PS-ASOs compared with PMOs are explained by differences in their protein binding [24,28].

The molecular mechanisms underlying the cellular uptake and intracellular transport of oligonucleotides are complex and insufficiently characterized, which poses a challenge to better understanding the distribution of oligonucleotide therapeutics. Because oligonucleotides are taken up into nonparenchymal cells (e.g., sinusoidal endothelial cells and Kupffer cells in the liver) or accumulated in the lumen of lysosomes of parenchymal cells, overall tissue concentrations may not be indicative of their effective concentrations in those tissues.

The cellular uptake process can be broadly divided into two steps, namely the adsorption of ASOs to cell surface proteins and internalization [16]. Various cell surface receptors have been reported to be involved in the receptor-mediated endocytosis of ASOs, including epidermal growth factor receptors, G-protein-coupled receptors, and scavenger receptors [31–33]. In particular, scavenger receptors have been extensively studied and found to mediate the uptake of naked ASOs, specifically stabilin-1 and stabilin-2 for the hepatic uptake of PS-ASOs [33] and scavenger receptor class A1 for the muscular uptake of PMOs [34].

Internalized ASOs need to be released from endosomes to interact with target RNAs in the cytosol or nucleus, and it is considered that these intracellular ASO trafficking processes are regulated by interactions with multiple proteins in the cytoplasmic and nuclear compartments. Using an affinity selection approach with biotinylated PS-ASOs, Liang *et al.* identified a set of intracellular proteins with which PS-ASOs interact [35]. A recent study showed that Rab5C and early endosomal antigen 1 (EEA1) in the early endosomal pathway, and Rab7A and lysobisphosphatidic acid in the late endosomal pathway, are involved in the trafficking of PS-ASOs and facilitate their endosomal escape after stabilin-mediated internalization [36]. Compared with PS-ASOs, there are fewer reports on the molecular mechanisms underlying the intracellular trafficking of PMOs.

Distribution studies for oligonucleotide therapeutics will therefore likely benefit from microscopic approaches, such as fluorescence imaging [37], mass imaging [38], and immunohistochemistry [39], to complement well-established approaches conventionally used for small molecule drugs. Autoradiography studies for oligonucleotide therapeutics are generally technically challenging and time consuming because oligonucleotide therapeutics are more difficult to radiolabel than small molecule drugs. Although ^14^C is often used to label small molecules, for ASOs with a larger molecular weight, the resulting molar specific activity after ^14^C labeling may be insufficient for sensitive detection and quantification. The random labeling of ASOs with tritium, which has a higher molar specific activity than ^14^C, may be an alternative, but there are some disadvantages to using tritium. Specifically, tritium can be re-exchanged with hydrogen, and quantitative interpretation is difficult when metabolized. Efforts have also been made to develop tag-based labeling methods, but it is necessary to confirm that the tag does not alter pharmacological activity and PK properties [40].

### Metabolism

Metabolic studies investigate the process of structural conversion of a drug and identify metabolites with potential efficacy or toxicity, including cross-species differences. In the case of small molecule drugs, metabolism primarily takes place in the liver and is thus often evaluated *in vitro* in hepatocytes, liver microsomes, and other liver preparations. After the study drug is incubated with an animal or human liver preparation, a sample of the resulting mixture is analyzed using LC/MS/MS or another analytical technique to evaluate metabolite stability, the kinetics of metabolic reactions, the structure of major metabolites, metabolic pathways, and interspecies differences in metabolism. Metabolism is generally evaluated *in vivo* by analyzing samples (e.g., plasma, urine, feces, and bile) from animals dosed with a radiolabeled drug using LC/MS/MS connected to a radioactivity detector to elucidate the structure and abundance of metabolites. The presence of a metabolite specific to humans requires further investigation of the relevance of that metabolite to efficacy, toxicity, and DDIs.

**FIG. 3.**
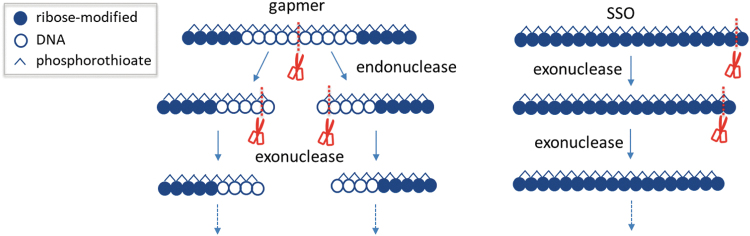
Schematic illustration of typical metabolic pathways of PS-ASOs.

In terms of metabolism of oligonucleotide therapeutics, PS-ASOs are hydrolyzed by endonucleases and/or exonucleases present in the plasma and tissues throughout the body. The stability of oligonucleotides against these nucleases and which site in the sequence is cleaved depends on the chemistry type of oligonucleotides (gapmer or SSO) and sugar modifications (Fig. 3). Gapmers, in which the central sequence of PS DNA nucleotides is flanked by sequences of sugar-modified residues at each end, are typically first cleaved by an endonuclease in the gap portion in the middle of the sequence, followed by exonuclease degradation [41]. SSOs, which contain sugar-modified residues throughout their sequence, are relatively stable and are gradually metabolized by exonucleases from the ends of the sequence [12]. PMOs, in contrast, exhibit much higher stability against metabolism [17].

Because the metabolic processes of oligonucleotide therapeutics mediated by nucleases are completely different from those associated with small molecule drugs, such as hepatic cytochrome P450 (CYP) oxidation and Phase 2 conjugation reaction, the materials and methodologies suitable for *in vitro* metabolic studies of oligonucleotide therapeutics have not been well established. Although the metabolic profiles of oligonucleotide therapeutics can be evaluated with a radiolabeled compound, as for small molecule drugs, the design of the labeling site and data analysis should take into account the possibility that the label can be removed as the compound is degraded into shortmers. It should also be noted that shortened metabolites from the ends may have pharmacological activity.

According to the guideline for preclinical safety assessment of oligonucleotide therapeutics issued by the Ministry of Health, Labour and Welfare in Japan, safety is not considered to be a particular concern for naturally occurring nucleic acid components degraded by nucleases; however, metabolites and degradation products containing chemically modified moieties need to be evaluated for nonclinical safety in accordance with ICH M3(R2), similar to conventional chemical products [42,43]. The Toxicokinetic and Pharmacokinetic Studies section in ICH M3(R2) makes the following recommendation: “The key assessment for nonclinical characterization of a human metabolite(s) is to identify metabolite(s) with exposures greater than 10% of total drug-related exposure and at significantly greater levels in humans than the maximum exposure seen in the toxicity studies” [42].

In the case of PS-ASOs, similar metabolic pathways for mice, monkeys, rats, and humans have been observed for several compounds [44], demonstrating that there is no significant species difference in the metabolic pathways of PS-ASOs and that systemic exposure of PS-ASO metabolites is generally low. For example, Post *et al.* reported comprehensive biotransformation of volanesorsen, a PS-ASO gapmer, in preclinical animal species and humans [45]. The plasma metabolite profiles of volanesorsen were similar across species, with volanesorsen as the major component. Various shortened metabolites (5–19 mer) were identified in the tissues and urine of mice and monkeys, with urinary metabolite profiles similar across species, including humans [45]. Based on currently available findings on the metabolism of ASOs, it is unlikely that there are unique or disproportionate ASO metabolites in humans that require safety evaluation under the guidance given above.

### Excretion

Excretion is evaluated to quantitatively determine how the parent drug and its metabolites are eliminated from the body. In general, for small molecule drugs, radioactivity in the urine, feces, bile, and expired air of animals dosed with a radiolabeled drug is measured over time with a liquid scintillation counter to determine the main routes of excretion and the amount excreted over time. The rates of excretion of the parent drug and major metabolites can also be measured using LC/MS/MS analysis of samples collected after an unlabeled drug is administered, although this requires chemically synthesized reference standards for quantification.

PS-ASOs are primarily excreted in the urine as the intact form and nuclease-shortened metabolites, whereas PMOs are excreted intact [24,28]. The excretion rate of PS-ASOs tends to be slower compared with PMOs because the rate of urinary excretion depends on the level of plasma protein binding, as discussed above. It is thus generally difficult to evaluate elimination profiles of PS-ASOs until completion of excretion. A long period (e.g., 3 months) may need to be set for excreta collection and the residual radioactivity measured in the carcass at the final observation point.

### Drug–drug interactions

DDI studies, in general for small molecule drugs, evaluate both the effects of the study drug on the pharmacokinetics of concomitant drugs (i.e., the study drug as a perpetrator of DDI) and the effects of concomitant drugs on the pharmacokinetics of the study drug (i.e., the study drug as a victim of DDI) [46]. Key molecules shown to affect the pharmacokinetics of small molecule drugs include drug-metabolizing enzymes, such as CYP and UDP-glucuronosyltransferase (UGT), as well as drug transporters, including organic anion transporting polypeptide and P-glycoprotein. Interactions caused by a study drug are evaluated using materials such as recombinant metabolizing enzymes, hepatocytes, and transporter-expressing cells to determine whether the drug causes the inhibition or induction of metabolism or whether it inhibits the transport of typical substrates. To investigate interactions in which the pharmacokinetics of the study drug are altered by concomitant drugs, the metabolizing enzymes and/or drug transporters that contribute substantially to the pharmacokinetics of the drug need to be identified by *in vitro* studies.

Like small molecule drugs, the DDIs associated with oligonucleotide therapeutics are evaluated both as a perpetrator and a victim of DDI based on the experimental approaches described above. It has been reported that oligonucleotide therapeutics do not (or only weakly) inhibit or induce drug-metabolizing enzymes and drug transporters and are not substrates of these enzymes and transporters [47–50]. Although PD-related mechanism-based interactions need to be considered (e.g., downstream or upstream effects of target gene knockdown on drug metabolizing enzymes or transporters), the DDI potential for oligonucleotide therapeutics using direct inhibition/induction of drug metabolizing enzymes and/or drug transporters is anticipated to be low.

A comprehensive study on *in vitro* DDI assessment of ASOs demonstrated that 2′-MOE-PS-ASOs did not significantly inhibit CYP1A2, CYP2B6, CYP2C8, CYP2C9, CYP2C19, CYP2D6, CYP2E1, or CYP3A4 in cryopreserved human hepatocytes at concentrations up to 100 μM ASO (half maximal inhibitory concentration [IC_50_] > 100 μM) [49]. In addition, CYP induction experiments with human cryopreserved hepatocytes demonstrated that 2′-MOE-PS-ASOs, at concentrations up to 100 μM, did not significantly increase the activity of CYP1A2 (0.723- to 1.44-fold), CYP2B6 (0.634- to 1.84-fold), or CYP3A4 (0.725- to 2.28-fold) [49]. That study also evaluated interactions with nine major transporters as per the recommendations of regulatory guidelines. None of the ASOs tested was a substrate for any of the transporters evaluated, with uptake <2-fold compared with controls, and efflux ratios <2.0 for breast cancer resistance protein and P-glycoprotein [49].

In another study, Kazmi *et al.* compared human liver microsomes (HLMs) to cryopreserved human hepatocytes for the *in vitro* assessment of DDI, including inhibition of CYP enzymes and UGT by PS-ASOs [51]. When HLMs were used for incubation, PS-ASOs exhibited direct inhibition of almost all CYP and UGT enzymes, with potent inhibition of CYP1A2 (IC_50_: 0.8–4.2 μM), CYP2C8 (IC_50_: 1.1–12 μM), and UGT1A1 (IC_50_: 4.5–5.4 μM). In contrast, in hepatocytes there was little to no direct inhibition of CYP by PS-ASOs, demonstrating that the results are dependent on the test system used [51]. As described in the Distribution section, only a limited fraction of ASOs can be taken up into cells and escape from endosomes, with the difference in the inhibitory effects of ASOs between hepatocytes and HLMs possibly explained by the concentration of ASOs to which the enzymes are exposed.

Because the processes and molecules involved in the metabolism and distribution of oligonucleotide therapeutics are different from those of small molecule drugs, it is necessary to discuss whether the rationale and approaches for evaluating DDIs associated with small molecule drugs are directly applicable to oligonucleotide therapeutics, particularly in terms of the choice of materials for *in vitro* experiments, how to set clinically relevant concentrations, and cutoff criteria in the decision tree.

### Bioanalysis

Concentrations of oligonucleotide therapeutics are determined not only with LC/MS/MS, which is generally used for small molecule drugs, but also with hybridization assays.

For LC/MS/MS analysis of oligonucleotide therapeutics, ion-pair chromatography using hexafluoro-2-propanol and triethylamine is primarily used for sample separation. Although the LC/MS/MS-based approach has the advantage of relatively fast method setup, it has the disadvantages of degrading the mobile phase and analytical columns, making long-term, robust analysis difficult [52–54].

In a typical procedure for hybridization assays, a template oligonucleotide that includes a sequence complementary to the analyte is immobilized on a solid surface and incubated with the analytical samples for hybridization, followed by ligation of a tagged probe sequence and then detection using enzyme-labeled antibodies [55–58]. The detection sensitivity of this procedure can be increased with the use of other detection methodologies, such as an electrochemiluminescent platform [59]. One of the key approaches to achieving ultrasensitivity in hybridization assays is signal amplification using branched DNA technology. A recent study established a branched DNA-based bioanalytical method for ASO quantification with adequate accuracy, precision, selectivity, and specificity, as well as acceptable matrix interferences [60]. This branched DNA assay showed significantly improved sensitivity (with a lower limit of quantification of 31.25 pg/mL in plasma), 6.4- and 16-fold higher than the sensitivity of a dual-probe hybridization assay with electrochemiluminescence and a single-probe hybridization ligand binding assay, respectively.

The hybridization assays enable highly sensitive analysis, but a method for quantification of parent compounds may also detect shortened metabolites such as (n–1) mer, so attention should be paid to the selectivity of the parent and metabolites. To overcome the drawbacks in specificity and sensitivity of the conventional hybridization assays and LC/MS/MS methods, an alternative bioanalytical method has recently been developed by combining hybridization-based sample pretreatment and LC-MS/MS-based detection [54]. This hybrid method enabled the robust quantitation of an ASO drug candidate in monkey serum, cerebrospinal fluid, and tissues in the range of 0.5–500 ng/mL [61].

Because each bioanalytical technique has its own pros and cons, it is important to choose a platform according to the purpose, nature of the samples, and stages of drug discovery and development.

### Delivery approaches

Various DDS approaches have been developed and used for oligonucleotide therapeutics. The major functions of DDS include: (i) targeted delivery to tissues; (ii) protection from degradation; and (iii) prolongation of exposure and pharmacological effects. There are two major types of approaches for DDS: chemical conjugation-based methods and carrier-based methods.

The conjugation of ASOs to a receptor ligand can facilitate the entry of ASOs into target cells and tissues. The most well-established of these systems is the asialoglycoprotein receptor (ASGPR)-mediated uptake of oligonucleotides conjugated to GalNAc, a natural ASGPR ligand, into liver hepatocytes. This technology has already been used in marketed siRNA as givosiran, lumasiran, inclisiran, and vutrisiran (Table 1), and its application to ASOs has been extensively studied [62–64]. ASGPRs are primarily expressed in hepatocytes, and their physiological function is to clear desialylated glycoproteins from the blood through clathrin-mediated endocytosis. Triantennary GalNAc-conjugated ASOs are efficiently tethered to the receptor and internalized in hepatocytes, resulting in an ∼10-fold increase in their potency *in vivo* [65].

Other examples of specific receptor targeting include the conjugation of glucagon-like peptide-1 receptor (GLP1R) agonist ligands as carrier peptides for targeted delivery of ASOs to pancreatic β cells [66]; a neurotensin receptor–ligand system for targeted delivery of ASOs to the CNS [67]; and transferrin receptor 1 (TfR1)-targeted delivery of ASOs to muscular tissues by conjugating them to a TfR1-binding fragment [68]. Recently, growing attention has been directed toward the conjugation of ASOs to lipid/fatty acid ligands. Conjugation of ASOs to palmitic acid increased plasma exposure and improved the delivery of ASOs to the interstitial space of mouse muscle, resulting in improved potency (three- to seven-fold) of target gene knockdown [69]. With regard to CNS delivery, DNA/RNA heteroduplex oligonucleotides (HDOs) conjugated to cholesterol or α-tocopherol were delivered to the brain, spinal cord, and peripheral tissues after subcutaneous or intravenous administration in mice and rats and suppressed target gene expression by up to 90% in the CNS [70].

Lipid nanoparticles (LNPs) are a representative example of carrier-based approaches. LNPs have been applied to siRNA and mRNA and put to practical use as patisiran (an RNAi therapeutic) and in vaccines for SARS-Cov-2 [71–73]. The application of LNP-based DDS for ASOs has also been studied for efficient and selective delivery to the liver [74,75]. It has been reported that the optimal LNP compositions for ASOs are different from those for siRNAs or mRNAs, probably due to differences in the key processes for successful delivery [76].

As described above, there has been significant progress in ASO delivery technology, particularly in targeting the liver, with extrahepatic delivery attracting considerable attention as a future challenge.

## Summary, Points to Consider, and Perspective

Table 3 summarizes the DMPK profiles of ASOs according to chemistry type and compares them with those of small molecule drugs. The DMPK/DDI profiles of small molecule drugs are highly diverse and depend on their structural and physicochemical characteristics (i.e., molecular weight, functional groups, lipophilicity, polar surface area) and can be characterized by well-established experimental approaches according to current guidelines. In contrast, oligonucleotide therapeutics share similar DMPK profiles within each chemistry type (e.g., PS-ASO and PMO), as highlighted in Table 3, and the impact of oligonucleotide sequences on these profiles is generally less significant. Most importantly, the factors that characterize the DMPK profile of oligonucleotides, especially the mechanisms and molecules involved in their distribution and metabolism, are different from those of small molecules. In addition, there are some points to consider regarding experimental approaches, such as bioanalytical challenges, the use of radiolabeled tracers, materials for *in vitro* metabolism/DDI studies, and methods to study biodistribution:

**Table 3.** Drug Metabolism and Pharmacokinetics Characteristics of ASOs Compared with Small Molecule Drugs

	ASOs		Small molecule drugs
	PS-ASO	PMO	
Absorption (plasma PK)	• Poor oral absorption due to low membrane permeability • High bioavailability after SC administration • Rapid decrease in plasma concentration primarily due to tissue distribution, showing biphasic plasma PK profiles		• Diverse oral absorption profiles depending on membrane permeability and solubility (formulation)
Distribution	• Accumulation in tissues with discontinuous/fenestrated capillaries, such as the liver and kidneys • Cellular uptake by receptor-mediated endocytosis		• Passive diffusion and/or transporter-mediated cellular uptake depending on chemical structure • Diverse protein binding depending on charge and lipophilicity
	• High plasma protein binding • Persistent tissue distribution	• Low plasma protein binding • Fast elimination from tissues	
Metabolism	• Hydrolyzed by nucleases present throughout the body • Metabolic profile depends on chemistry type and sugar modifications	• Highly stable against metabolism	• Diverse metabolism through oxidation, conjugation, and/or hydrolysis depending on chemical structure and physicochemical properties
Excretion	• Gradually excreted in the urine as the intact form and shortmers	• Rapidly excreted in the urine as the intact form	• Biliary and/or urinary excretion depending on chemical structure
DDI	• No or only weak inhibition or induction of drug-metabolizing enzymes and drug transporters • Not substrates of drug-metabolizing enzymes or drug transporters		• Diverse DDI potentials via drug metabolism and/or transport depending on chemical structure
Bioanalysis	• (Ion-pair) reverse phase LC/MS/MS and/or hybridization assays are used		• Reverse-phase LC/MS/MS is mostly used

- ASOs are taken up into nonparenchymal cells or are accumulated in lysosomes, and thus, overall tissue concentrations may not be indicative of efficacy.
- In addition to conventional distribution studies (e.g., autoradiography) used for small molecule drugs, microscopic approaches, such as fluorescence imaging, mass imaging, and immunohistochemistry, are beneficial in analyzing the cellular and subcellular distribution of oligonucleotides.
- Radiolabeling of ASOs is generally more difficult compared with small molecules, and attention should be paid to the position of the label because terminal labels can be metabolically removed.
- The materials and methodologies suitable for *in vitro* metabolic studies of ASOs are still not well-established; it is important to profile shortmers from the ends because they may have pharmacological activity.
- Highly sensitive bioanalytical methods need to be developed to determine low plasma concentrations of ASOs in the elimination phase as a surrogate of tissue concentrations.
- In addition to LC/MS/MS analysis, hybridization assays enable highly sensitive analysis, but a method for quantification of parent compounds may also detect short-chain metabolites such as (n–1) mer, so attention should be paid to the selectivity of the parent and metabolites.
- Because the excretion of PS-ASOs is slow, it is difficult to evaluate elimination profiles of PS-ASOs until completion of excretion; a longer period for the collection of excreta and measurement of residual radioactivity in the carcass at the final observation point should be considered.
- Because the molecules involved in the ADME of ASOs are different from those of small molecules, DDI assessment approaches for small molecule drugs (e.g., the choice of materials for *in vitro* experiments, how to set clinically relevant concentrations, and cutoff criteria in the decision tree) may not be directly applicable to ASOs.
- Targeted delivery approaches for ASOs, including chemical conjugation-based methods and carrier-based methods, have been extensively studied. The ADME profiles and evaluation strategies of ASOs can be significantly affected by the nature of the delivery system used.

A working group of the International Consortium for Innovation and Quality in Pharmaceutical Development (IQ consortium) has recently published a recommendation article regarding protein binding and DDI of siRNA [77]. In addition, the Japan Pharmaceutical Manufacturers Association task force for DMPK evaluation of oligonucleotide therapeutics and a regulatory science research group for oligonucleotide therapeutics in the Japan Agency for Medical Research and Development collaboratively performed a comprehensive survey on DMPK evaluations of marketed ASO therapeutics [78]. Active discussion among industry, regulators, and academia will bring about optimal and efficient approaches to evaluate the DMPK/DDI profiles of oligonucleotide therapeutics.
